# A Perspective on the Use of NB-UVB Phototherapy vs. PUVA Photochemotherapy

**DOI:** 10.3389/fmed.2018.00184

**Published:** 2018-07-02

**Authors:** Sally H. Ibbotson

**Affiliations:** Photobiology Unit, Dermatology Department, Ninewells Hospital, University of Dundee School of Medicine, Dundee, United Kingdom

**Keywords:** UVB, PUVA therapy, phototherapy, skin diseases, psoriasis, eczema, vitiligo

## Abstract

Narrowband UVB (NB-UVB) phototherapy and psoralen-UVA (PUVA) photochemotherapy are widely used phototherapeutic modalities for a range of skin diseases. The main indication for NB-UVB and PUVA therapies is psoriasis, and other key diagnoses include atopic eczema, vitiligo, cutaneous T-cell lymphoma (CTCL), and the photodermatoses. The decision on choice of phototherapy is important and NB-UVB is usually the primary choice. NB-UVB phototherapy is a safe and effective therapy which is usually considered when topical agents have failed. PUVA requires prior psoralen sensitization but remains a highly effective mainstay therapy, often used when NB-UVB fails, there is rapid relapse following NB-UVB or in specific indications, such as pustular or erythrodermic psoriasis. This review will provide a perspective on the main indications for use of NB-UVB and PUVA therapies and provide comparative information on these important dermatological treatments.

## Introduction

Narrowband UVB (NB-UVB) phototherapy and psoralen-UVA (PUVA) photochemotherapy are widely used light-based treatments for a range of diverse skin diseases and can be highly effective, well-tolerated, safe, cost-saving, and reduce the need for topical therapies (1–6). The main indication for NB-UVB or PUVA is psoriasis (7) but other mainstay indications include atopic dermatitis or dermatitis of other cause, vitiligo, cutaneous T-cell lymphoma (CTCL), and a range of other conditions, including the photodermatoses, pityriasis rubra pilaris, urticaria, aquagenic pruritus, urticaria pigmentosum, pityriasis lichenoides, lichen planus, granuloma annulare, alopecia areata, and graft vs. host disease (2, 3, 5, 6) (Table 1).

**Table 1:** Key indications for NB-UVB or PUVA.

Psoriasis
Pustular or erythrodermic^*^
Eczema – atopic or other type
Vitiligo
Cutaneous T-cell lymphoma
Patch
Plaque^*^
Photodermatoses
Polymorphic light eruption, actinic prurigo, solar urticaria, hydroa vacciniforme, erythropoietic protoporphyria
Chronic actinic dermatitis*
Urticaria
Urticaria pigmentosa
Aquagenic pruritus
Mastocytoses
Generalised pruritus
For example secondary to cholestasis or uraemia
Pityriasis lichenoides chronica
Lichen planus
Granuloma annulare
Graft vs. host disease
Alopecia areata^*^
Pityriasis rubra pilaris^*^
Hand & foot eczema^*^
Palmoplantar pustulosis^*^

* *Consider PUVA in preference to UVB*.

If topical treatments fail to establish adequate control of disease then a light-based therapy would be a next appropriate treatment choice and in most instances NB-UVB would be selected as the primary phototherapeutic option. However, in certain diseases such as erythrodermic or pustular psoriasis, pityriasis rubra pilaris, or plaque stage CTCL, PUVA would be the desired option (5).

I am going to provide my opinion and perspective on the relative uses of NB-UVB and PUVA for a range of diseases, with particular emphasis on psoriasis as the predominant indication for a UV-light based therapy and with briefer mention on the salient points relative to the use of NB-UVB and PUVA in other conditions. I am restricting my review to NB-UVB and PUVA and am not including BB-UVB or UVA1 phototherapies.

## Background

UVB was introduced into increasingly widespread and routine use following developmental work in the 1980s (8–11). NB-UVB phototherapy reduces the need for topical therapies (1) and is a cost effective (12) and safe treatment, which involves repeated controlled delivery of the narrowband region of the UVB spectrum centered on 311 nm (4, 6). The main acute adverse effects of NB-UVB are erythema and induction of photosensitivity diseases, such as polymorphic light eruption (PLE). However, although the risk of erythemal episodes may be increased by concomitant phototoxic drugs (13, 14), this can be minimized by undertaking a baseline minimal erythema dose (MED) and establishing treatment protocols based on an individual's MED (15). This also allows any unsuspected abnormal photosensitivity diseases to be detected, in particular solar urticaria or chronic actinic dermatitis (CAD). Induction of PLE may occur during a treatment course but generally can be accommodated via dose adjustments and judicious use of topical corticosteroid, without the need to stop NB-UVB (16). Other uncommon side-effects, such as psoriatic lesional blistering, occasionally occur but generally treatment is very well-tolerated (17, 18). Importantly, NB-UVB can be safely used in children and in pregnancy and long-term studies to date do not indicate a significantly increased risk of skin cancer over an age- and sex-matched control population who have not received UVB phototherapy (19–21).

PUVA photochemotherapy is delivered using psoralen administration via either systemic (8-methoxypsoralen or 5-methoxypsoralen) or topical (usually now 8-methoxypsoralen as bath, soak, gel, cream, or lotion) routes (5). The mechanism of action of PUVA is quite distinct from that of UVB or of UVA alone, with PUVA inducing a delayed erythemal reaction peaking around 96 h after irradiation of psoralen-sensitized skin (22–27). This contrasts with the peak time for development of erythema after NB-UVB exposure of 12–24 h (28). Treatment is thus logistically slightly more of a challenge as psoralen sensitization is required. With systemic PUVA, appropriate skin and eye protection must be used for 24 h after psoralen ingestion. Oral 8-methoxypsoralen may cause some gastrointestinal upset, although switching to 5-methoxypsoralen minimizes this adverse effect and of course this is not an issue with topical PUVA. However, PUVA treatment can be highly effective and very safely administered in any Dermatology Department with a significantly sized Phototherapy Unit.

With the exception of less common adverse effects such as PUVA pain, treatment is otherwise usually well-tolerated (5). Undoubtedly, there is a longer term risk of skin carcinogenesis with high numbers of PUVA exposures (19, 29–37), but the risks can be minimized by vigilance, limitation of lifetime numbers of PUVA exposures, and avoidance of the use of maintenance PUVA where possible. As with all therapeutic approaches, benefit, and risk must be evaluated and it is important that PUVA is kept firmly in the range of treatment options as it can be highly effective, resulting in clearance, and marked improvement in quality of life for patients with psoriasis and a variety of other diseases.

It is essential that adequate governance is ensured for the safe delivery of both NB-UVB and PUVA therapies. In Scotland we have established the National Managed Clinical Network for phototherapy (Photonet; www.photonet.scot.nhs.uk), which employs a central database (Photosys), enabling standardization of treatment protocols, recording of treatment parameters, and outcomes and facilitating linkage studies to ascertain longer-term risks of treatment, notably skin cancer risk (20, 21). This has been an invaluable asset to allow standardization of phototherapy services in Scotland and delivery of effective and safe treatment for patients. This approach is now being adopted in England and has important roles in delivery of optimized safe care.

## Psoriasis

The main indication for any light-based therapy is psoriasis, and for the reasons highlighted in terms of practicalities and ease of treatment and its safety and potential for use in children and pregnancy, NB-UVB phototherapy would usually be the light-based therapy of choice, with high clearance rates achieved for chronic plaque psoriasis (6, 38–40).

In an initial controlled comparative half-body study in 10 patients with widespread psoriasis, no significant difference in efficacy was seen between twice weekly NB-UVB or systemic PUVA (41) and this observation was also reported in a separate intra-individual open non-randomized controlled paired comparison study of three times weekly NB-UVB and PUVA, with no significant difference in efficacy seen between the treatment arms. However, there was a trend to superior efficacy with PUVA and this was particularly evident for patients with a higher baseline PASI score (42), possibly suggestive of a role for PUVA in more severe psoriasis or relapsing psoriasis, although given the convenience of NB-UVB this would generally be the preferred initial approach. In a separate inter-individual study of 100 patients with psoriasis, twice weekly PUVA was superior in efficacy to twice-weekly NB-UVB, with 35% of patients still being clear at 6 months after completion of PUVA, compared with only 12% after NB-UVB (43). These findings are supported by those of a separate study in which 93 patients with chronic plaque psoriasis were randomized to receive either twice-weekly oral PUVA or twice-weekly NB-UVB, resulting in 84% achieving clearance with PUVA compared with significantly lower clearance rates (65%) with NB-UVB and shorter remission, as 6 months after treatment 68% of those treated with PUVA were still in remission, compared with only 35% of patients treated with NB-UVB (44). Of note, lower clearance rates were achieved in patients of skin phototype V and VI, with only 24% achieving clearance, although baseline psoriasis severity was not a determinant of response in this study (44). However, high efficacy rates have been reported in patients of higher skin phototypes (IV and V), with 81–82% of patients showing marked improvement with three times weekly 8-MOP PUVA or NB-UVB and no difference between the two treatment regimens, indicating that phototherapy or photochemotherapy should certainly still be considered for patients with higher skin phototypes (45).

Given that three-times weekly NB-UVB results in faster more efficient clearance of psoriasis than twice-weekly treatment (46), comparison of twice weekly PUVA with a twice-weekly NB-UVB regimen is likely to be including a sub-optimal NB-UVB treatment arm. Indeed, in an intra-individual randomized controlled study of three times weekly NB-UVB with twice-weekly TMP bath PUVA, NB-UVB was of superior efficacy and also resulted in more rapid response of psoriasis, with 75% clearance compared with 54% with PUVA (40). Additionally, in a randomized intra-individual half-side study in patients with chronic plaque psoriasis, comparing three times weekly TMP bath PUVA and three times weekly NB-UVB, again NB-UVB was of superior efficacy compared with TMP bath PUVA, although all patients relapsed within 4 months of follow-up (47). In contrast, Salem et al., undertook a randomized controlled trial in 34 patients, comparing 8-MOP bath PUVA three times a week with three times weekly NB-UVB and greater reduction in PASI score was seen with PUVA than NB-UVB, along with greater reduction in peripheral CD4+ T Cells, indicative of possible systemic effects (48). Furthermore, Markham et al., undertook an open randomized inter-individual comparative study of twice-weekly oral 8-MOP PUVA with three times weekly NB-UVB for chronic plaque psoriasis and showed equivalent efficacy in terms of time to clearance and period of remission (49).

Thus, trying to make sensible conclusions from this diverse range of study findings, given the ease, convenience, and safety of treatment and the study evidence, NB-UVB should usually be considered as the first phototherapeutic option for patients with chronic plaque psoriasis, with PUVA used when NB-UVB is not effective or there is rapid relapse once NB-UVB is discontinued (39). A lower threshold for considering PUVA is reasonable if psoriasis is particularly thick and/or extensive at baseline, including erythrodermic and pustular psoriasis (50) or the patient is of higher skin phototype. In addition, 8-MOP bath or oral PUVA may be preferable to TMP bath PUVA, as although no head to head comparison has been undertaken, lower response rates are reported for those studies using TMP bath PUVA rather than 8-MOP (40, 47–49). Erythemogenic doses of PUVA are not a pre-requisite for clearance (51) and maintenance PUVA or NB-UVB for psoriasis should generally be avoided (52). Failure to respond to NB-UVB does not equate to prediction of a lack of response to PUVA and the latter should be considered for those who fail to do well with NB-UVB. For children, NB-UVB phototherapy is preferred and PUVA is relatively contraindicated, although this is not an absolute rule, but given the concerns about long-term safety, PUVA would not be the first line choice.

## Eczema

Whilst any light-based treatment approach is less straightforward for eczema than psoriasis, not least for the reason of flaring of eczema in the early stages of treatment mainly due to the heat load of therapy, both NB-UVB and PUVA can be highly effective for the treatment of atopic eczema and other forms of eczema (5, 6). However, the evidence-base is relatively weak and there are no prospective studies comparing head-to-head systemic PUVA with NB-UVB (53). Systemic 5-MOP PUVA was shown to be superior to medium dose UVA1 for atopic eczema in an intra-individual randomized controlled comparison study (54). Bath PUVA can also be highly effective for atopic eczema (55). Bath PUVA using 8-MOP was compared with NB-UVB in a small half-side comparison study, showing that both were effective for severe atopic eczema without a significant difference between the two therapies (56). Thus, NB-UVB would usually be the first line of choice for atopic eczema, given the ease of administration, safety, and potential for use in children (57). Given the response of atopic eczema to several types of light-based therapy and if NB-UVB phototherapy fails or there is early relapse after discontinuation of treatment, then the options of either PUVA or UVA1 exist, although given the lack of evidence of superiority of UVA1, the latter would likely only be considered if PUVA was contraindicated. Indeed, a combination of NB-UVB and UVA or UVA1 could be considered for some patients, although whether this is advantageous compared with UVB alone is unclear and this needs further study (58).

## Vitiligo

For the treatment of vitiligo, NB-UVB has been shown to be superior to PUVA with respect to rates of repigmentation, particularly for unstable extensive vitiligo, and in achieving more cosmetically acceptable even repigmentation (59–63). Thus, NB-UVB would be the phototherapy of choice for vitiligo, although PUVA may be considered in certain cases, particularly if there is lack of response to NB-UVB.

## Cutaneous T-cell lymphoma

Whilst there are no direct head-to-head controlled trials of NB-UVB and PUVA for early stage CTCL, both have been shown to be effective for this stage of disease (5, 64). In one retrospective study 81% of patients with early stage CTCL achieved complete remission with NB-UVB, compared with 71% with PUVA (*n* = 56) (65). This observation has also been supported by two other studies showing equivalent efficacy for NB-UVB and PUVA in achieving remission of early stage CTCL (66, 67) and thus NB-UVB should be the phototherapy of choice for early patch stage CTCL disease, with complete remission in approximately three quarters of patients being achievable, although duration of remission has not been thoroughly evaluated and relapse may occur within 6 months (68). It is unclear whether phototherapy has any impact on limiting natural disease progression. Based on one study it was suggested that tumor stage CTCL was slower to develop and overall survival was improved in those who had previously received phototherapy, although given the retrospective nature of the study these data must not be obver-interpreted (69). For thicker plaque stage CTCL, the increased depth of penetration of PUVA is desirable and NB-UVB would not be indicated, whereas PUVA would be the phototherapeutic modality of choice (5). For tumor stage disease, PUVA as monotherapy would not suffice and combination therapy is likely to be required. Maintenance PUVA should generally be avoided, but occasionally is justified for maintenance use in CTCL (5, 70). However, other adjunctive agents should be considered and combination with retinoids, rexinoids, or interferon may be required or the use of radiotherapy for localized tumor stage disease or total skin electron beam treatment for more extensive involvement (5). Photopheresis may of course be required for Sezary syndrome (71, 72). Thus, in summary NB-UVB for early stage disease and PUVA for plaque stage disease as monotherapy or in combination therapy for more advanced disease should be considered as mainstays in management (5, 64, 73).

## The photodermatoses

There is a relative lack of randomized controlled trial evidence investigating the use of NB-UVB and PUVA for the abnormal photosensitivity conditions. However, for desensitization of PLE, comparative studies show equivalent efficacy for NB-UVB and PUVA (16). As regular annual desensitization courses may be required from a relatively young age, NB-UVB is preferred for PLE as the phototherapy of choice, although PUVA should be considered for treatment failures and when reported its use may be for more severe PLE (74, 75). Induction of PLE during treatment is common and to be expected but does not usually require early termination of the desensitization course and can usually be accommodated with reduction of dose increments and topical corticosteroid use during the treatment course (16, 76). With the other less common photodermatoses, desensitization phototherapies with either NB-UVB or PUVA may be considered and appropriate but will depend on the action spectrum for induction of abnormal photosensitivity and thus which light-based treatment approach can be tolerated. In general, these patients should be investigated and managed through a specialist photodermatology unit as there may be additional needs, such as inpatient requirements for suppression and light-protected care and advice regarding subsequent natural sunlight top up exposure. In CAD, the action spectrum for induction of abnormal photosensitivity is usually maximal in the UVB region and therefore NB-UVB phototherapy cannot often be tolerated. In this setting PUVA may need to be considered, sometimes in combination with topical superpotent or systemic corticosteroids in order to reduce the risk of disease flare, particularly in the early stages of treatment (77, 78).

NB-UVB and PUVA may also be useful therapeutic approaches for the other photodermatoses, such as erythropoietic protoporphyria, hydroa vacciniforme, actinic prurigo, and idiopathic solar urticaria (79). Indeed, in solar urticaria the action spectrum for induction of urticaria is usually in the UVA and visible parts of the spectrum and NB-UVB responses are typically normal, in which case NB-UVB desensitization can be used successfully for desensitization, with UVA rush hardening and/or PUVA considered if NB-UVB is not feasible or successful (79–84).

It would generally also be advisable for patients with solar urticaria to have anti-histamine cover whilst receiving a UV-based therapy. In EPP, as photosensitivity is maximal in the visible part of the spectrum, NB-UVB is usually well-tolerated and can be highly effective and is the phototherapy of choice. Whilst here is limited evidence to support the use of PUVA, given that patients with EPP will usually require annual treatment courses from a young age, NB-UVB is advised and PUVA is rarely justified (85–88). Similarly, whilst there is limited evidence to support the use of NB-UVB and PUVA in actinic prurigo, again given the young age and need for annual treatment, NB-UVB is advised and PUVA rarely needed, although may occasionally be required (79). Factors such as the age of the patient, risk factors such as skin phototype and evidence of photodamage and the action spectrum for induction of abnormal photosensitivity, should always be taken into account in any decision regarding NB-UVB or PUVA and for the photodermatoses, specialist advice regarding timing of desensitization courses, risk of induction of the condition by treatment and management of that, top-up exposure requirements after treatment and the need for annual treatment courses must be addressed in order to establish the optimal approach for any given patient.

## Localized hand and foot disease

Hand and foot dermatoses are a mixed group of conditions, which include hyperkeratotic eczema, psoriasis, psoriasiform dermatitis, palmoplantar pustulosis. There is a lack of robust evidence regarding the optimal management of these diseases, including the role of NB-UVB and PUVA therapies and there is no reason to consider that one approach will suit all conditions. Undoubtedly, NB-UVB and PUVA photochemotherapy may be useful for localized hand and foot dermatoses (89). Although oral PUVA and NB-UVB may both be effective for eczema of the palms and soles, oral PUVA has been shown to be superior to NB-UVB in two small studies from the same group, although relapse rates were high following both treatments (5, 90, 91). The depth of penetration of 8-MOP systemic PUVA may be desirable for recalcitrant hand and foot dermatitis and other uncontrolled studies have also shown high levels of efficacy with oral PUVA for hand and foot eczema (5, 92, 93). In contrast, topical PUVA has not been shown to be superior to placebo or any other active treatment, despite uncontrolled studies, and anecdotal observations that efficacy can be achieved and this is an area requiring further research. Thus, for hand and foot eczema, oral PUVA would be the light-based therapy of choice (5). Psoriasis of the palms and soles has been even less well evaluated and, whilst there is some evidence to support the use of PUVA, either with oral or topical psoralens, the strength of evidence is weak and further studies are required (5, 7, 94). For palmoplantar pustulosis, again oral PUVA either as monotherapy or combined with retinoids, may be highly effective (5, 95–97) and the role of NB-UV is less clear as has not been evaluated.

## Other indications

There is evidence that NB-UVB and PUVA may be effective for urticaria and indeed randomized controlled trial evidence to show the superior efficacy of NB-UVB plus anti-histamine compared with anti-histamine alone (98–100). More recently, superiority of NB-UVB compared with PUVA has been shown for urticaria (101), and thus NB-UVB should be considered as a treatment option if antihistamines and other pharmacological therapies fail and may provide useful disease remission. A range of other conditions may be effectively treated by NB-UVB and PUVA and include pityriasis lichenoides (102), granuloma annulare (103, 104), urticaria pigmentosa and cutaneous mastocytoses (105–107), aquagenic pruritus (108–110), lichen planus (111–114), alopecia areata (115–118), generalized pruritus, such as secondary to uraemia or cholestasis (119, 120), and graft vs. host disease (2, 3, 5, 6) and these phototherapeutic modalities may be invaluable treatment approaches for these otherwise difficult-to-treat groups of diseases. For conditions such as pityriasis rubra pilaris, which may be aggravated and flared by the use of NB-UVB, 8-MOP systemic PUVA should be considered.

## Conclusions

To summarize, NB-UVB phototherapy and PUVA photochemotherapy are both invaluable treatments to have available in any dermatology department and should be prioritized, not only for psoriasis, but in a variety of other inflammatory and proliferative skin diseases, including atopic eczema. Treatment can be safely and easily administered and is well tolerated with few adverse effects. Excellent disease remission may be achieved, whilst sparing the use of other potentially toxic drugs at a relatively early stage in a patient's journey. Head-to-head comparative monotherapy studies with biologic therapies do not exist and are needed. Due to the relative cost-efficacy of the phototherapies and the understanding of their long-term safety profiles compared with the cost and less lengthy follow-up for the biologics, these should be employed prior to consideration of biologic treatments (1). As with any therapy, standardization of optimized treatment regimens, careful observation of treatments delivered and therapeutic outcomes, adverse effects and long-term follow-up studies, including determining any skin cancer risk, are essential. The development of the National Managed Clinical Network for Phototherapy has had a major impact on standardization, safety, and vigilance in delivery of our phototherapy practices in Scotland and has proved to be an invaluable tool, enabling the place of NB-UVB, and PUVA therapies to continue to be well-established in the treatment of skin disease.

## Author contributions

The author confirms being the sole contributor of this work and approved it for publication.

### Conflict of interest statement

The author declares that the research was conducted in the absence of any commercial or financial relationships that could be construed as a potential conflict of interest. The reviewer FL and the handling Editor declared their shared affiliation.
